# Supplementary material for: Spatially resolved transcriptomics reveals the architecture of the tumor-microenvironment interface
Source: Nat Commun. 2021 Nov 1;12:6278. doi: 10.1038/s41467-021-26614-z (PMC8560802; doi:10.1038/s41467-021-26614-z)
Supplement: Supplementary file 1 — Supplementary Information [file 41467_2021_26614_MOESM1_ESM.pdf]

Supplementary Information

Spatially resolved transcriptomics reveals the architecture of the tumor-microenvironment interface

Miranda V. Hunter, Reuben Moncada, Joshua M. Weiss, Itai Yanai, Richard M. White

Contents:

Supplementary Figures 1-14
Supplementary Tables 1-3

Supplementary Figure 1. Spatially resolved transcriptomics data statistics.
a-b. Violin plots showing number of transcripts (UMIs) detected per Visium array spot (a) and number of unique genes detected per spot (b). **c.** Images of the H&E stained tissue sections used for SRT. Scale bar, 2 mm. **d-e.** Numbers of UMIs and genes detected per spot, plotted onto the tissue array. **f.** Violin plot showing the number of UMIs per spot across the tissue types in the individual SRT samples. **g.** Approximation of the number of nuclei per Visium array spot (55 μm diameter) in the tumor and muscle regions (*n* = 10 images quantified for each).

Supplementary Figure 1

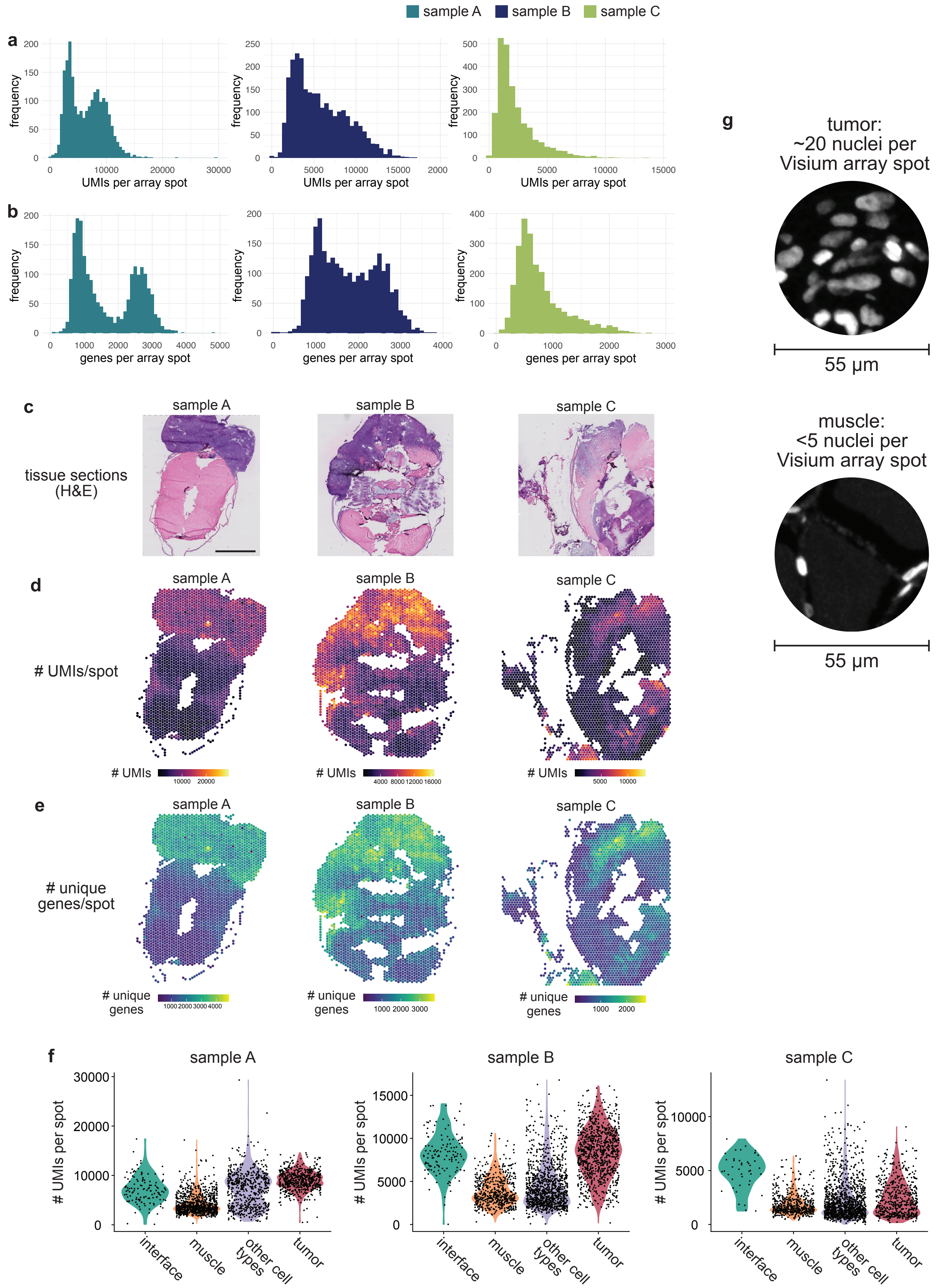

Supplementary Figure 2

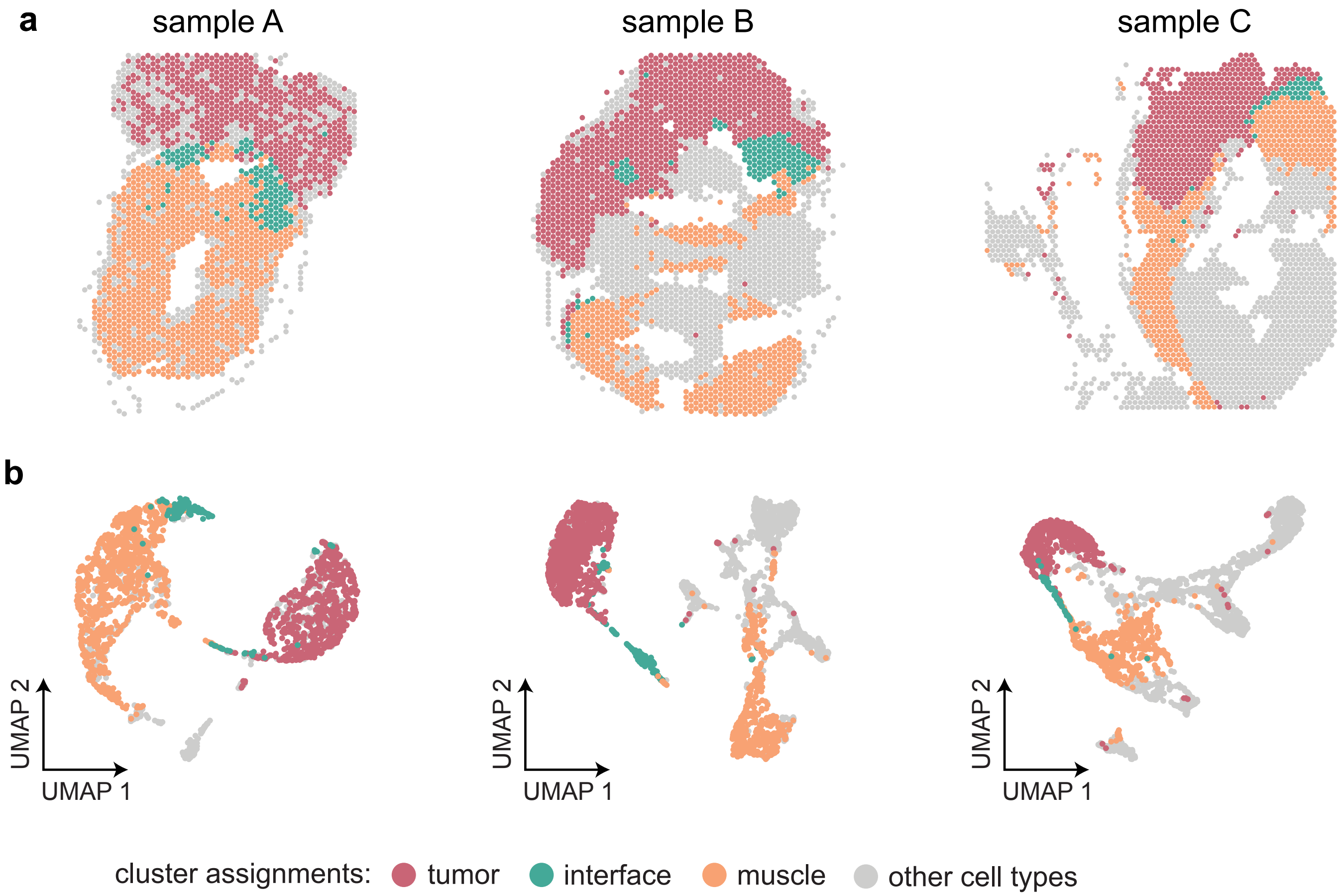

Supplementary Figure 2. A unique interface cluster is found in each SRT sample.
a. Cluster assignments plotted onto the Visium array for individual samples. b. Cluster assignments labelled in UMAP space for individual samples.

Supplementary Figure 3

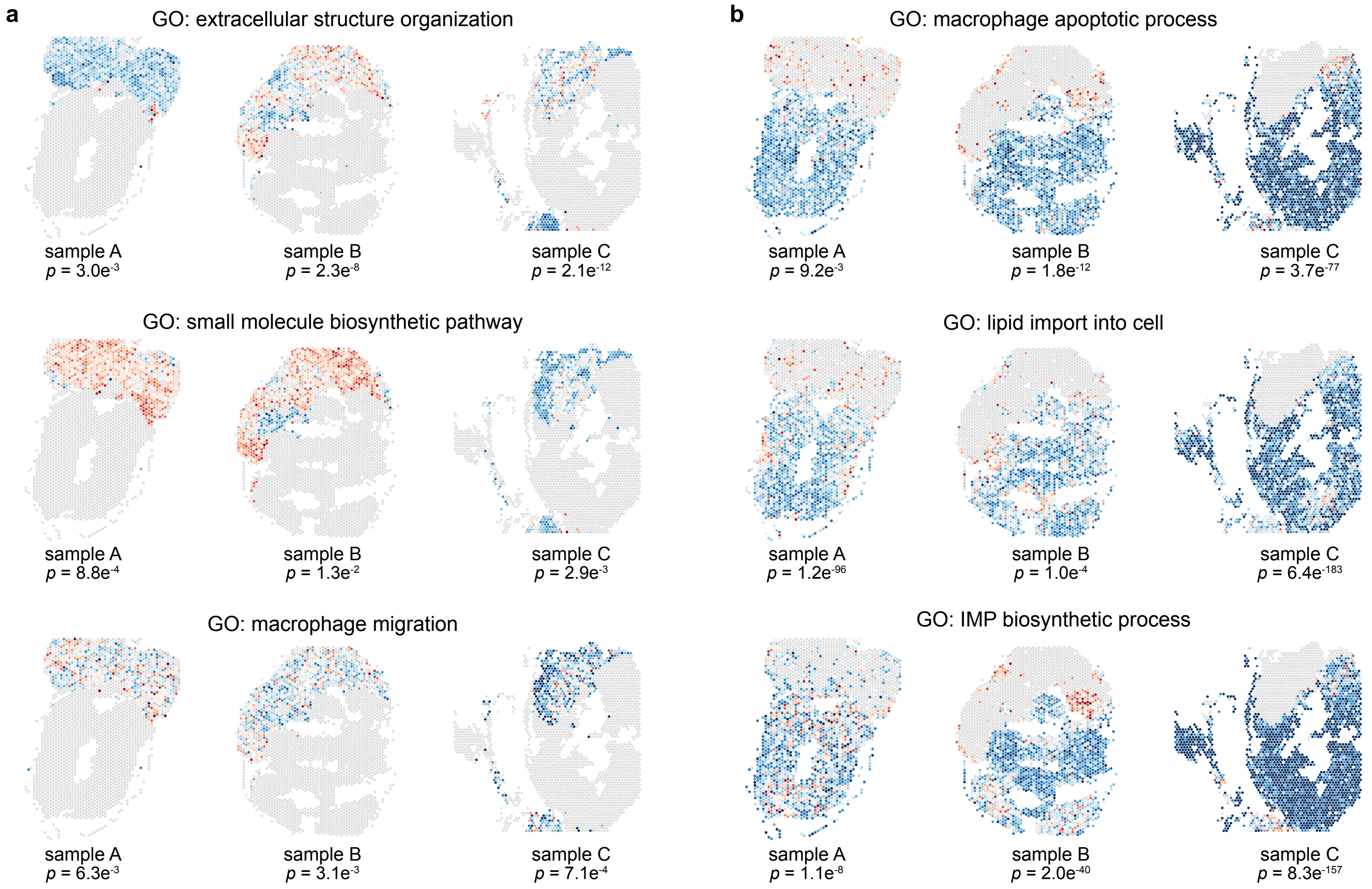

Supplementary Figure 3. Spatial patterning of biological pathways in the tumor and microenvironment.
a-b. Average, standardized expression of annotated genes for gene ontology (GO) terms displaying spatially-coherent expression patterns in the tumor (a) and microenvironment (b) regions in each SRT sample. P-values represent the comparison between the distance between spots expressing that GO term genes and a null-distribution of distances between random spots (Wilcoxon's rank sum test, two-sided, with Bonferroni's correction).

Supplementary Figure 4

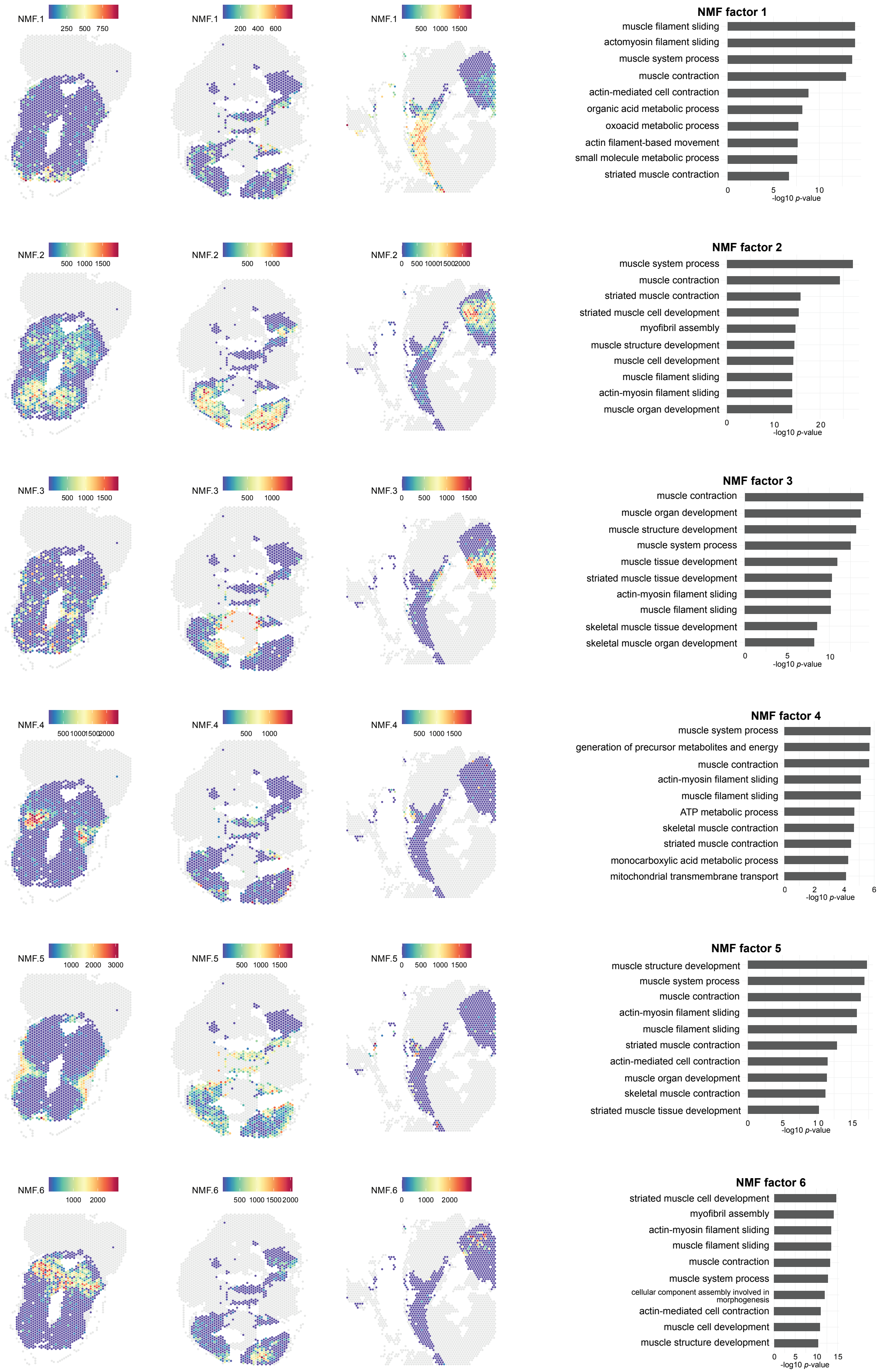

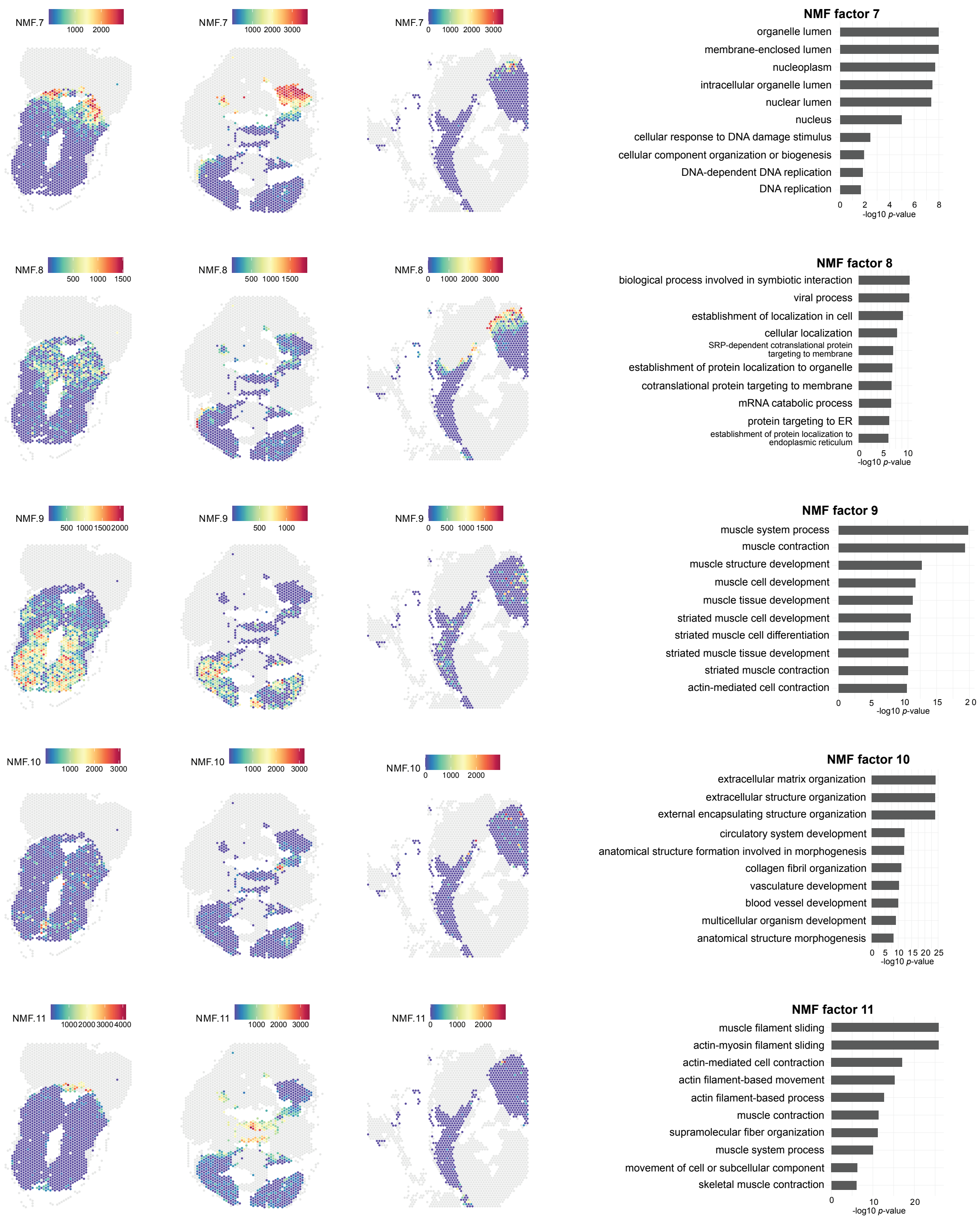

Supplementary Figure 4. Non-negative matrix factorization (NMF) analysis on SRT spots, and enriched GO terms for top genes in each NMF factor.
Non-negative matrix factorization (NMF) was performed on the integrated expression matrix of the three SRT samples, with k = 11 factors. Factor scores were then projected onto spots, and the top 150 scoring genes per factor were used for GO enrichment analysis. P values were calculated using the hypergeometric test (one-tailed).

Supplementary Figure 5. single-cell RNA-seq data statistics. a,d. Histograms showing the number of UMIs (left) and genes (right) per cell for each scRNA-seq reaction. **b,e.** Violin plots showing the number of UMIs (left) and genes (right) per cell for the different clusters in each scRNA-seq reaction. P-values were calculated using the Wilcoxon rank sum test (two-sided) with Bonferroni's correction. **c,f.** Cluster assignments for each scRNA-seq reaction plotted in UMAP space. **g.** Predicted possible doublets within the integrated scRNA-seq dataset.

Supplementary Figure 5

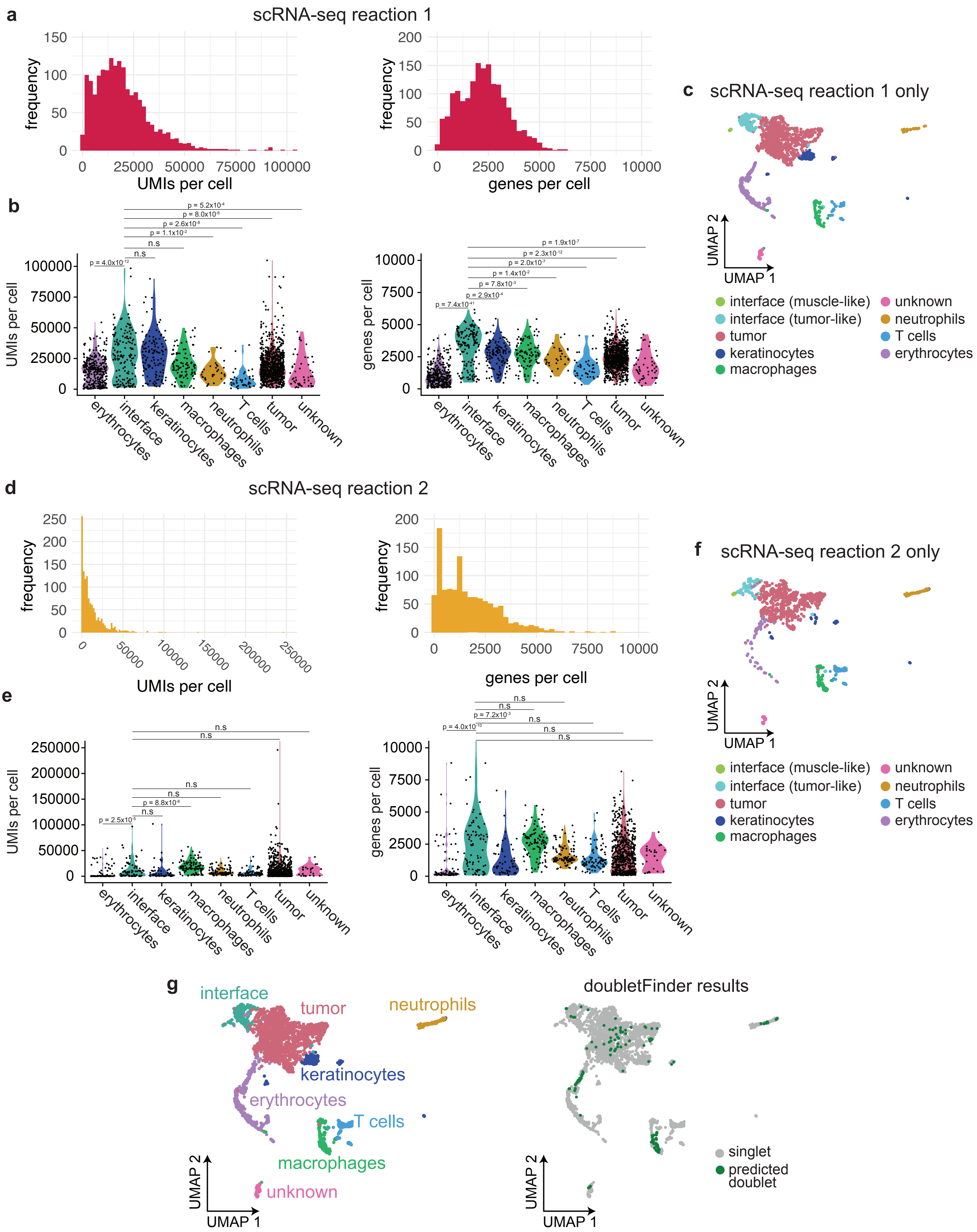

Supplementary Figure 6. single-nucleus RNA-seq data statistics. a. Histograms showing the number of UMIs (left) and genes (right) per nucleus. **b.** Violin plots showing the number of UMIs (left) and genes (right) per nucleus across the clusters in the snRNA-seq dataset. P-values were calculated using the Wilcoxon rank sum test (two-sided) with Bonferroni's correction. **c-d.** Mean expression per cell/nucleus for all common genes in the scRNA-seq and snRNA-seq dataset (15,022 genes) (c) and for the fish orthologs of the published SYSCILIA genes (320 genes).

Supplementary Figure 6

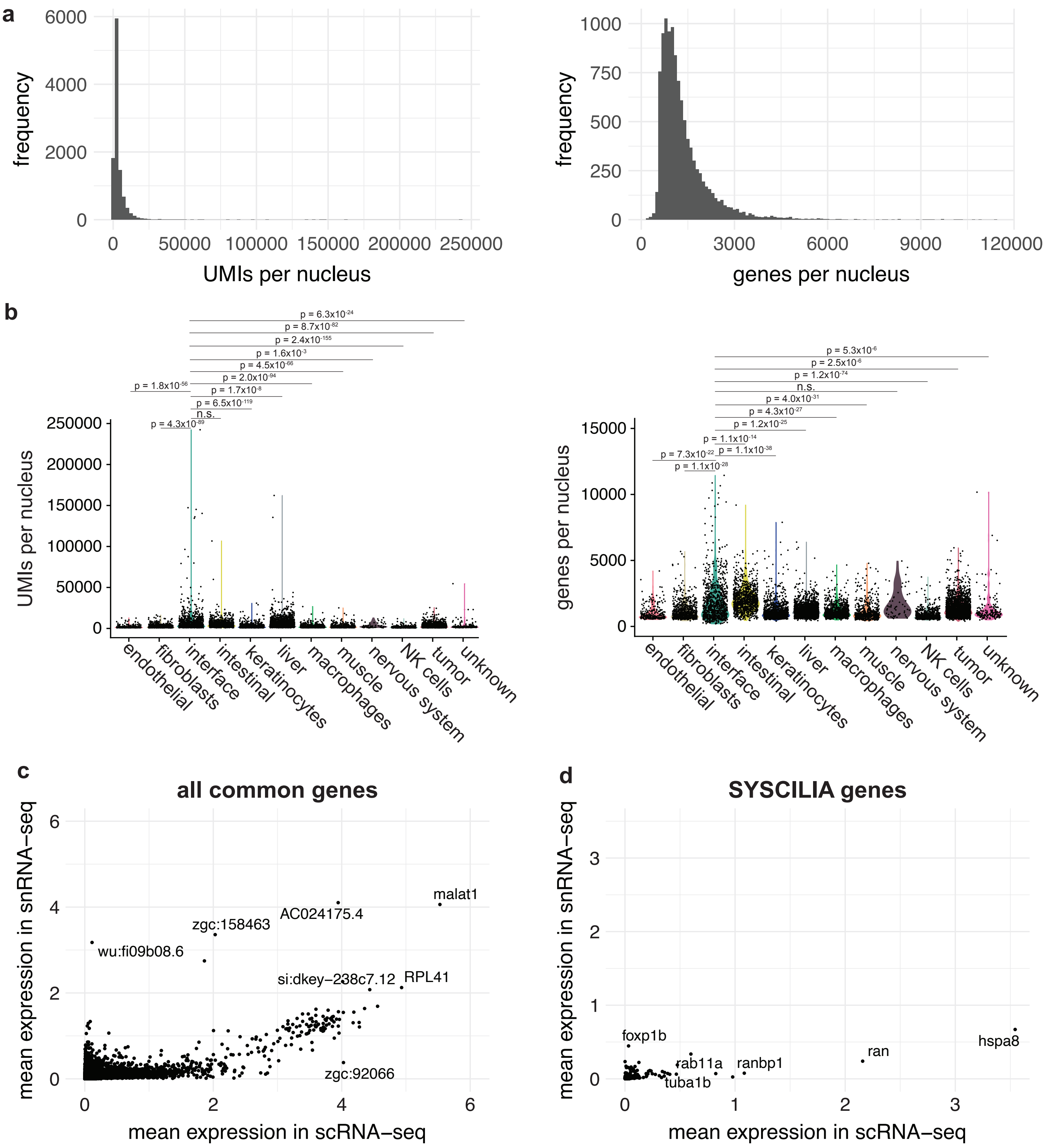

Supplementary Figure 7. Expression of cell-type specific genes within the snRNA-seq interface cell subclusters. (left) Overlap between genes significantly upregulated in the tumor/microenvironment cell clusters, and genes significantly upregulated in the corresponding interface subcluster. (right) Scoring of each nucleus for the proportion of genes upregulated in the tumor/microenvironment cell cluster. P-values calculated using the Wilcoxon rank sum test (two-sided).

Supplementary Figure 7

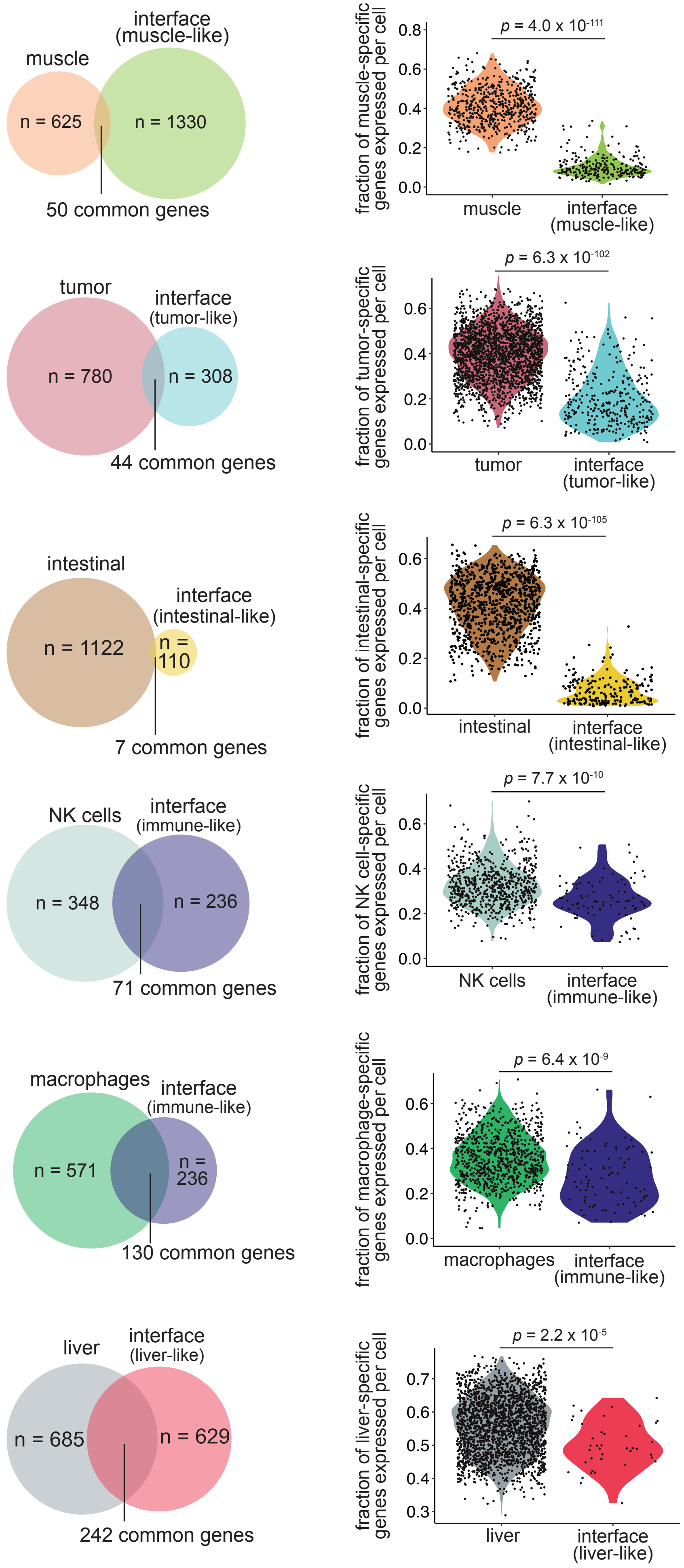

Supplementary Figure 8. Expression of cell-type specific genes from tumor, microenvironment and related interface clusters. Clusters are labelled on the top colorbar and marker genes are labelled on the left and right axes. Scaled (Z-scored) expression is shown.

Supplementary Figure 8

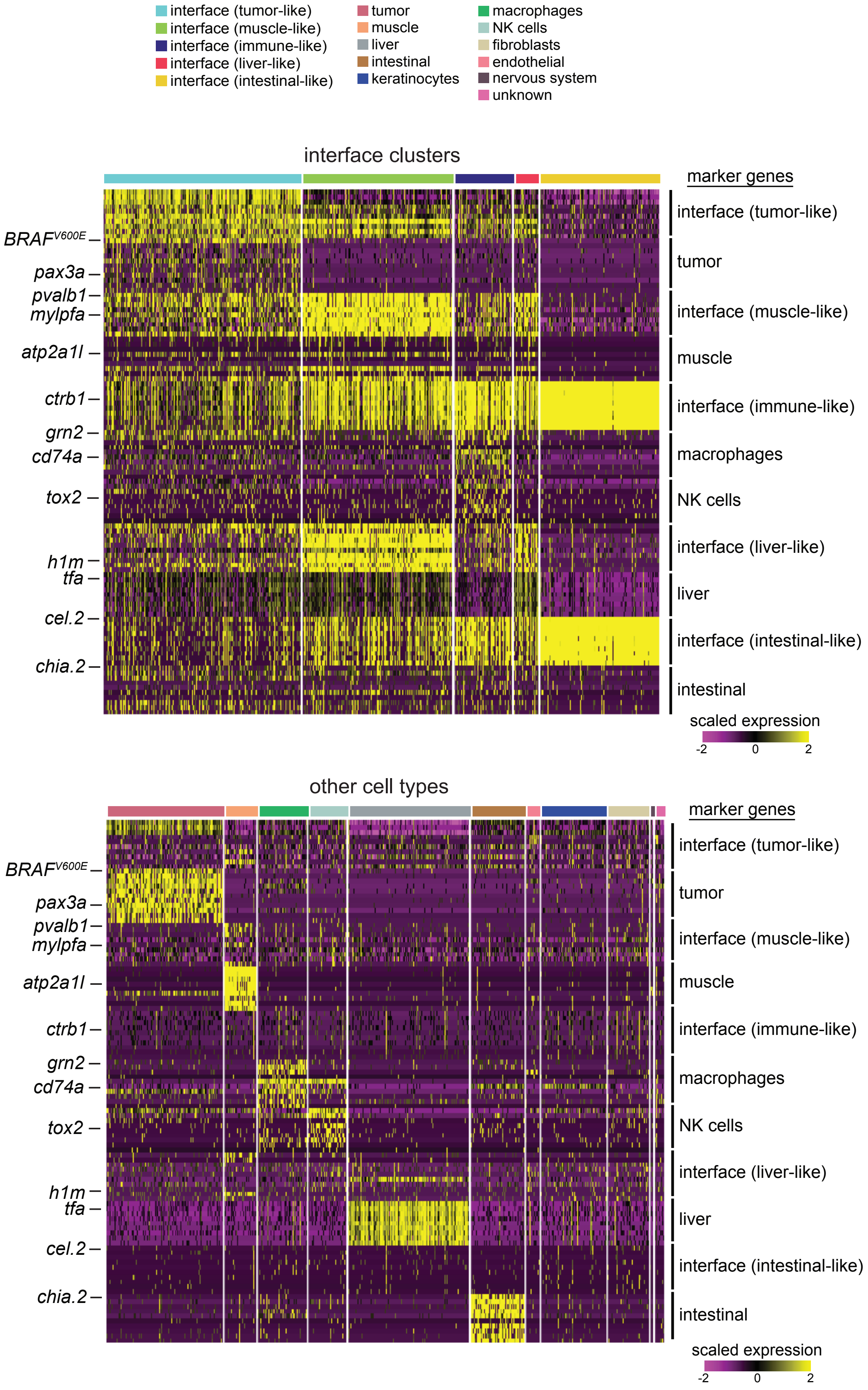

Supplementary Figure 9

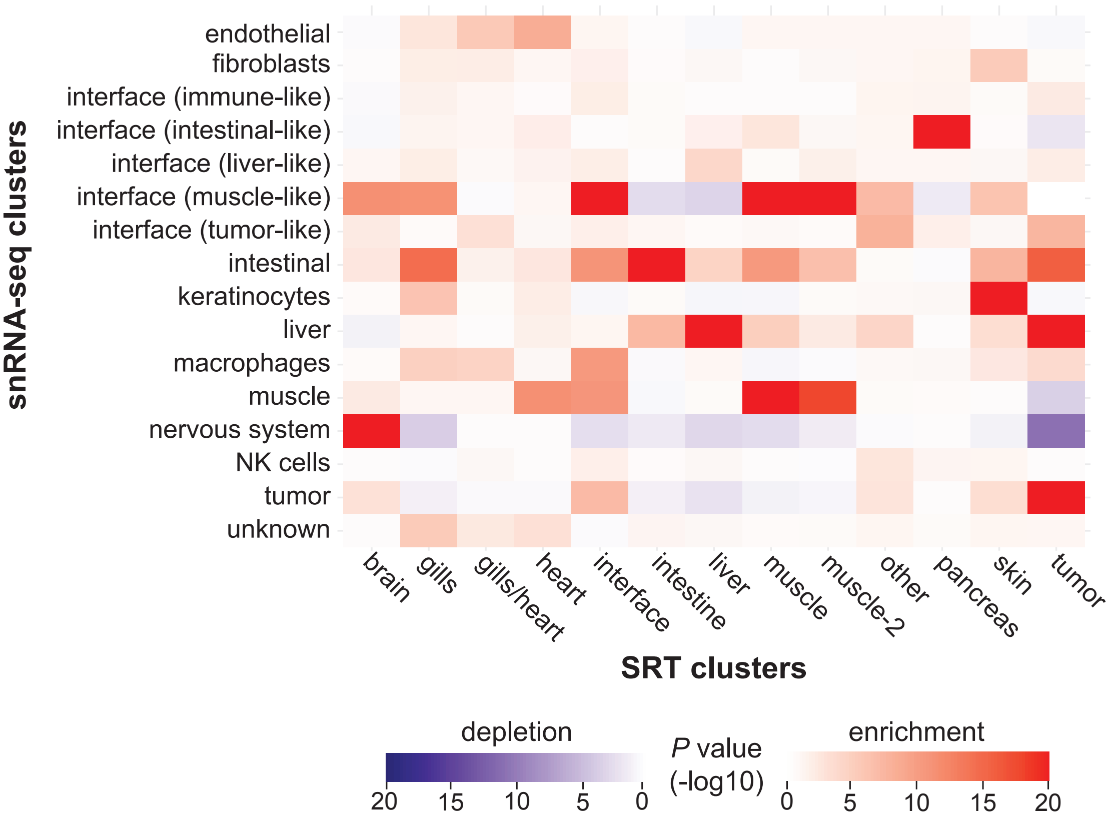

Supplementary Figure 9. Deconvolution of the SRT interface region using multimodal intersection analysis (MIA). MIA map for all snRNA-seq clusters compared to all SRT clusters. P-values were calculated using the hypergeometric test (one-tailed).

Supplementary Figure 10. Computational modeling of interface cell interactions with NicheNet. a. Predicted ligands expressed by interface cells, ranked by predicted ligand activity (Pearson correlation coefficient). **b.** Predicted receptors expressed by non-interface cells for the predicted ligands in **a. c.** Predicted target genes for the ligands in **a**. **d-e.** Normalized expression of the zebrafish orthologs of *HMGB2* (*hmgb2a* and *hmgb2b*) and *CDH1* (*cdh1*) in the snRNA-seq, scRNA-seq and SRT clusters.

Supplementary Figure 10

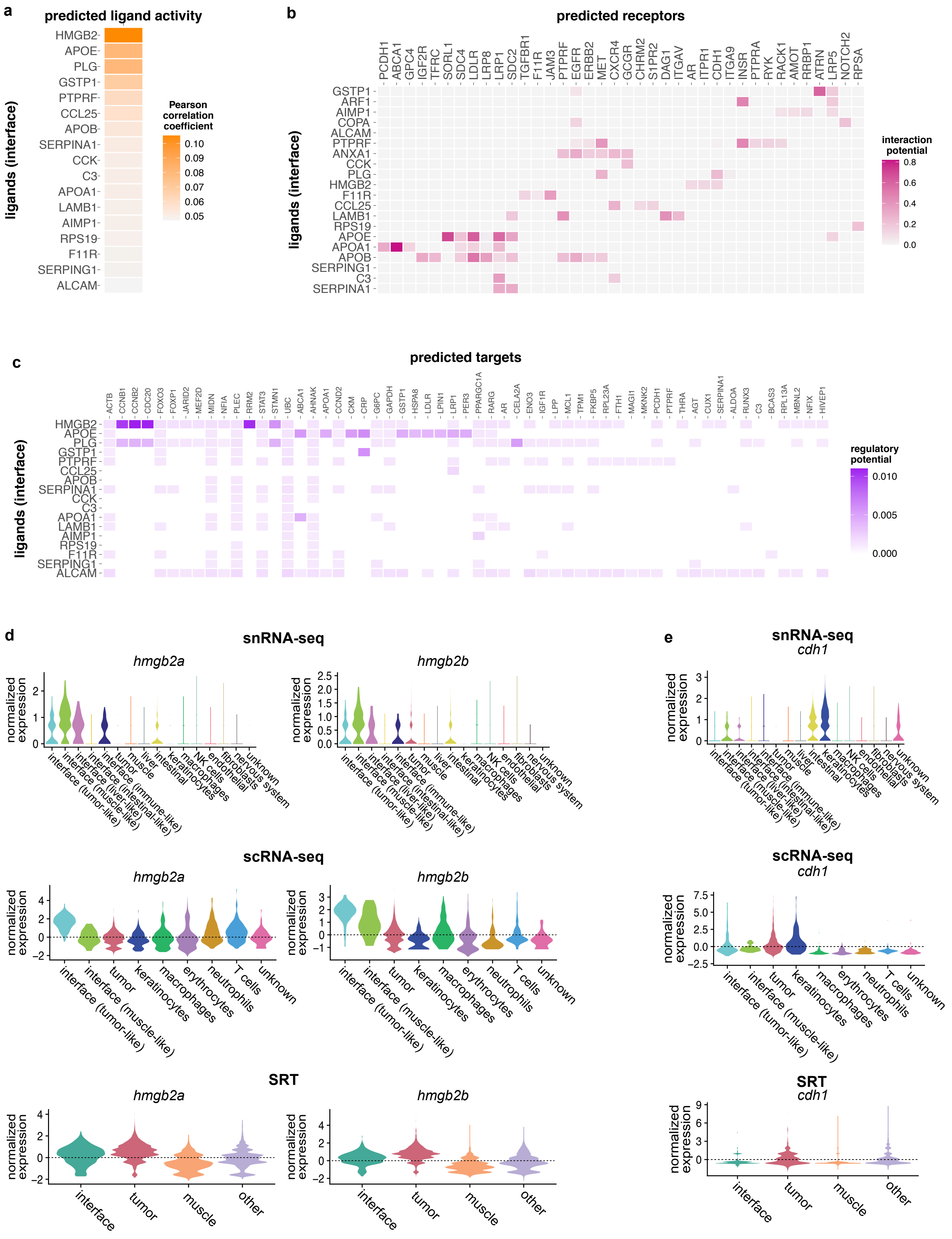

Supplementary Figure 11. A common interface gene signature shared between the scRNA-seq, snRNA-seq, and SRT datasets. Heatmaps showing expression of the common genes upregulated in the scRNA-seq, snRNA-seq, and SRT interface clusters. Full genelist can be found in **Table S2**. Genes are plotted in the same order across all three heatmaps.

Supplementary Figure 11

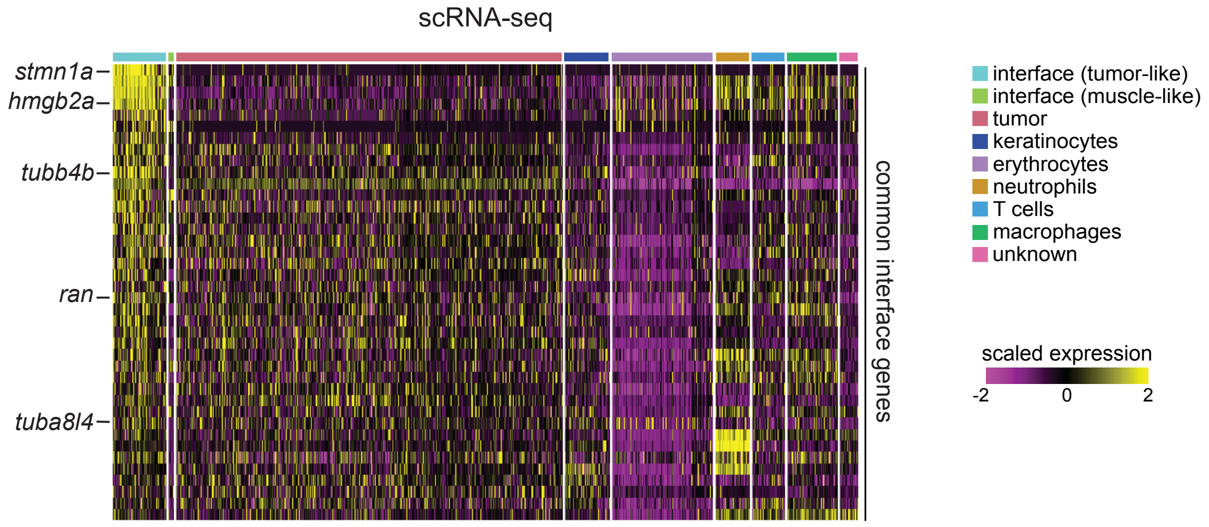

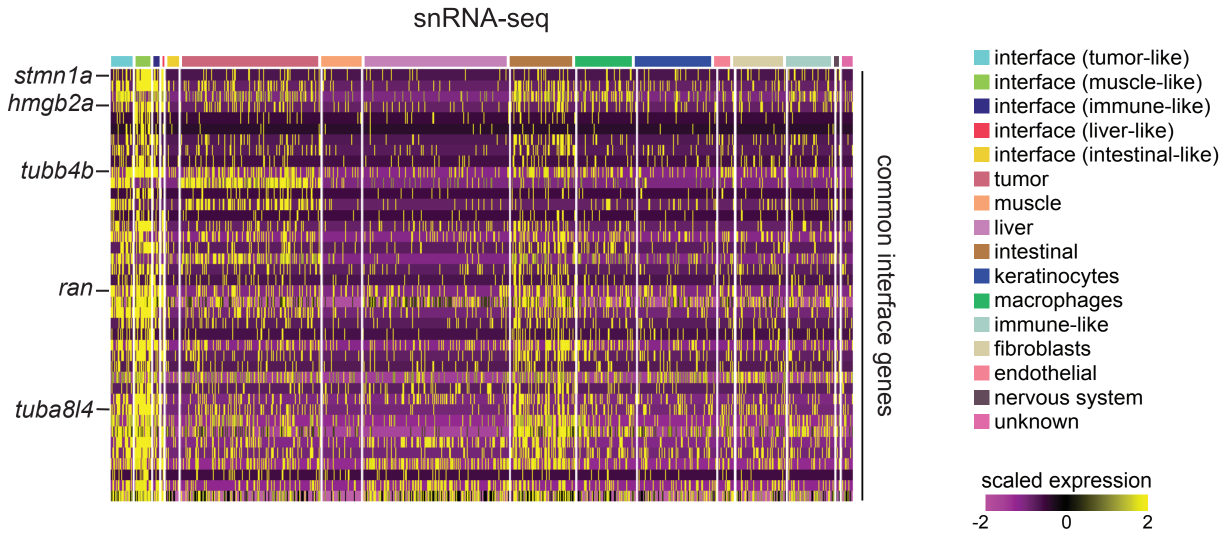

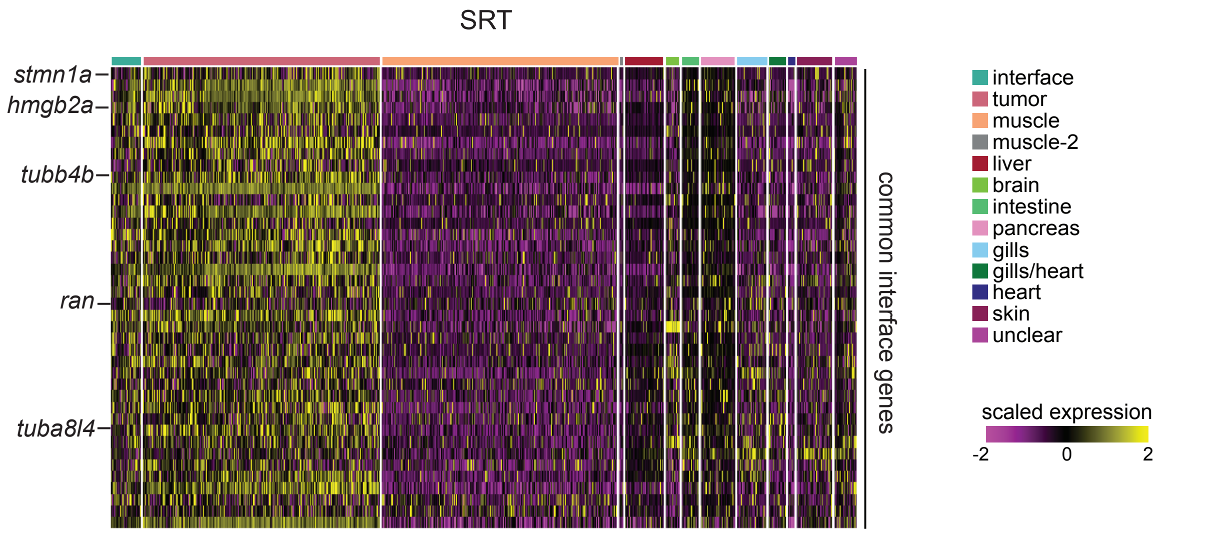

Supplementary Figure 12. Acetylated tubulin staining within the tumor, muscle, and tumor-muscle interface regions. Immunofluorescence images of tumor (green), Hoescht (blue) and acetylated tubulin (magenta) intensity at the tumor-muscle interface (top), center of tumor (middle) and distant muscle (bottom). Scale bars, 100 µm (40X magnification). Representative images are shown from *n* = 3 independent experiments.

Supplementary Figure 12

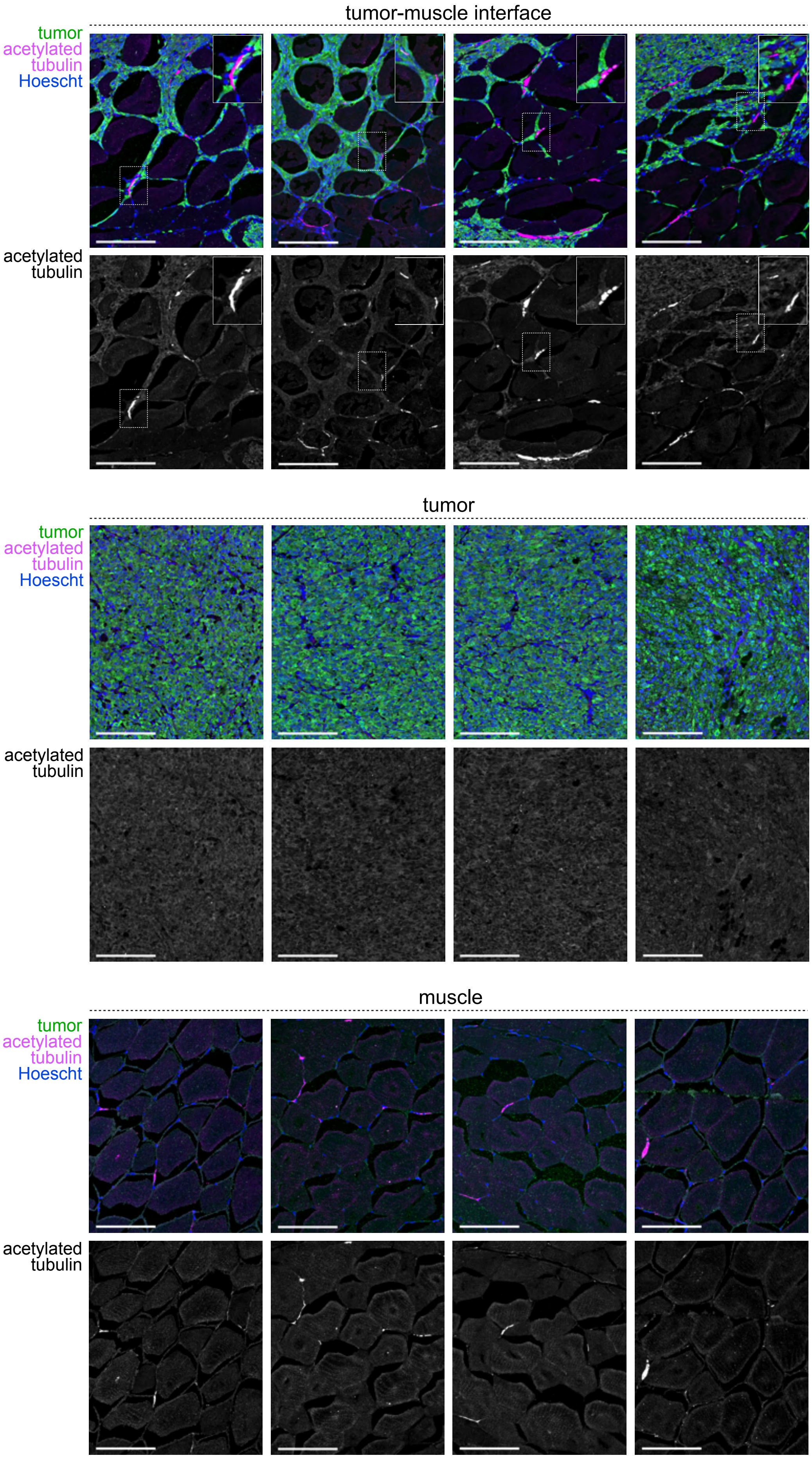

Supplementary Figure 13

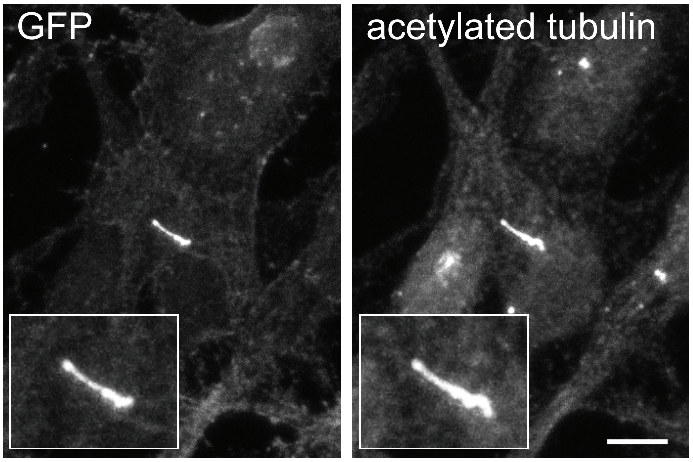

Supplementary Figure 13. Cilia are occasionally present on cultured zebrafish melanoma cells.
Staining of cultured ZMEL1 cells expressing a transgenic cilia reporter (ARL13B-GFP), stained for GFP (left) and the cilia marker acetylated tubulin (right). Scale bar, 5 µm. Representative images are shown from n = 3 independent experiments.

Supplementary Figure 14. An interface-like cell state may be present in human melanoma. a-c. UMAP projection of human metastatic melanoma scRNA-seq data from Smalley et al., 2021. Cluster assignments (a), interface marker gene expression score (b), and interface classification (c) are indicated. The cutoff for classification as an interface-like cell state is indicated in (b). **d.** Interface gene expression scores across the different clusters in the dataset. **e.** Normalized expression of the interface-specific genes *PLK1*, *HMGB2*, *TUBB4B*, and *TPX2*. **f.** SYSCILIA gene expression score across the 3 main interface cell states relative to the corresponding tumor/TME cell type. **d,f.** *p*-values were calculated using the Wilcoxon rank sum test (two-sided) with Bonferroni's correction as needed. **g.** Heatmap displaying scaled expression of interface, tumor, T/NK cell, and myeloid marker genes across all cells.

Supplementary Figure 14

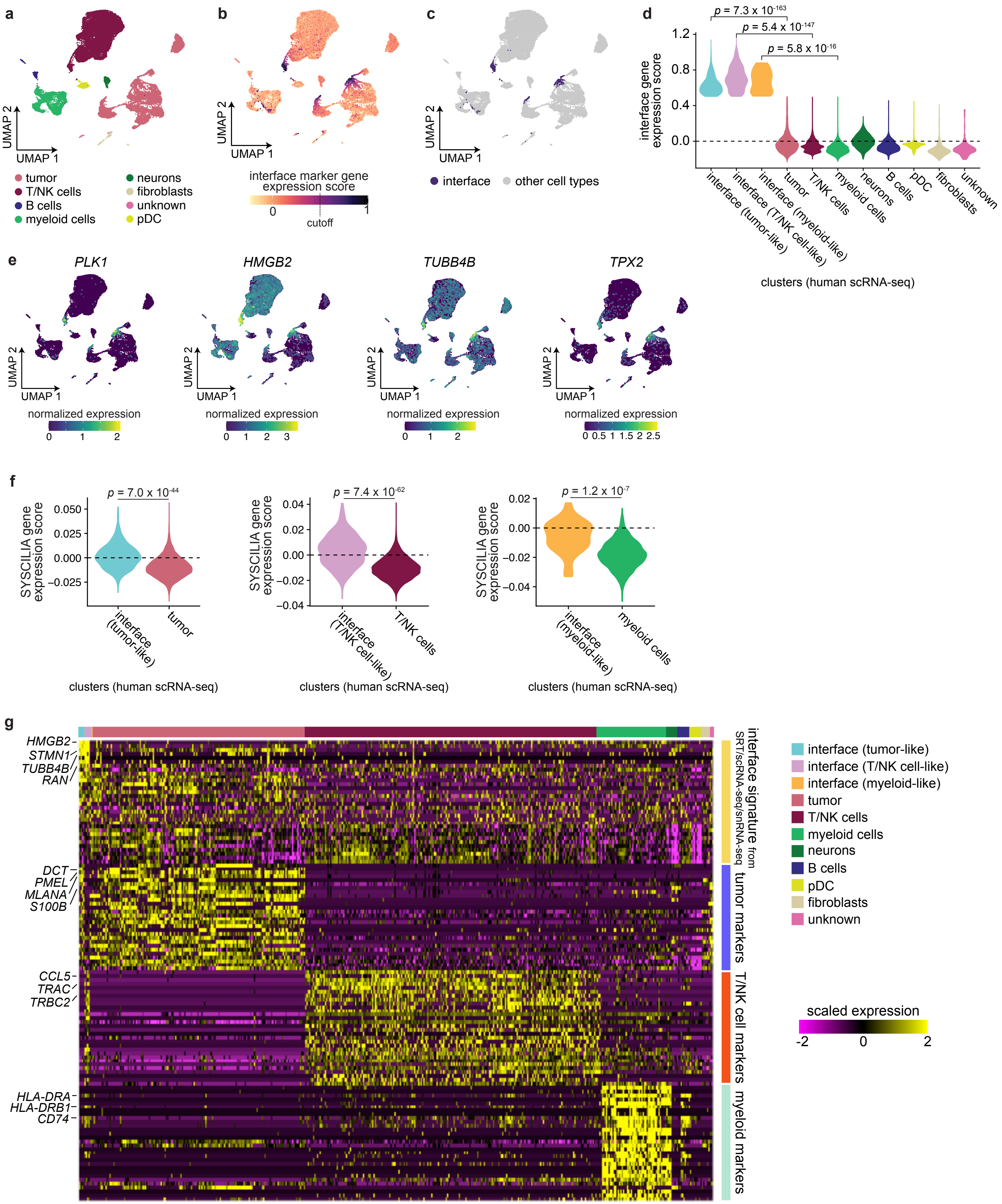

Supplementary Table 1

Common interface gene signature across the SRT, scRNA-seq and snRNA-seq datasets. Genes were calculated using the Wilcoxon rank sum test (two-sided).

**common interface genes**
stmn1a
h2afvb
hmgn2
hmgb2a
pcna
rrm2
hmga1a
snrpd1
nutf2l
tubb4b
BRAFhuman
sumo3a
zgc:123068
snrpe
hint1
cirbpa
snrpb
zgc:152791
fkbp1aa
snrpd2
ran
ppiab
ckbb
psmb6
cst14a.2
zgc:56493
cox6a1
psma1
khdrbs1a
hspe1
dad1
tuba8l4
cfl1
actb1
gstp1
zgc:153867
mibp2
si:dkey-188i13.11
eif3f
ppiaa

Supplementary Table 2

Activator/repressor status for the human ETS genes. Activator/repressor status and fish ortholog(s) were obtained from Uniprot and ZFIN.

Human gene	Fish ortholog(s) (Source: ZFIN)	Activator or repressor? (Source: Uniprot)
ERF	*erf, erfl1, erfl3*	Repressor
ELK1	*elk1*	Activator
ELK3	*elk3*	Either
ELK4	*elk4*	Either
ERG	*erg*	Unknown
FLI1	*fli1, fli1rs*	Activator
FEV	*fev*	Repressor
ETS1	*ets1*	Activator
ETS2	*ets2*	Activator
ETV2	*etsrp*	Activator
GABPA	*gabpa*	Activator
ETV5	*etv5a, etv5b*	Activator
ETV1	*etv1*	Activator
ETV4	*etv4*	Activator
SPDEF	*spdef*	Activator
EHF	*ehf*	Either
ELF3	*elf3*	Either
ELF4	*elf1*	Activator
ELF2	*elf2a, elf2b*	Either
ETV6	*etv6*	Repressor
ETV7	*etv7*	Repressor
SPI1	*spi1b*	Activator
SPIC	*spic, spicl1*	Activator

Supplementary Table 3

List of primers used in this study

Primer name	Sequence (5'-3')
MH027	GCCGCCCCCTTCACCATGTTCAGTCTGATGG
MH028	gcccttgctcaccatTGAGATCGTGTCCTGAGCATCACC
MH029	atggtgagcaagggcgag
MH030	GGTGAAGGGGGCGGC
