# Supplementary material for: Spatially resolved transcriptomics reveals the architecture of the tumor-microenvironment interface
Source: Nat Commun. 2021 Nov 1;12:6278. doi: 10.1038/s41467-021-26614-z (PMC8560802; doi:10.1038/s41467-021-26614-z)
Supplement: Supplementary file 3 — Description of Additional Supplementary Files [file 41467_2021_26614_MOESM3_ESM.docx]

Description of Additional Supplementary Files

**Title: Supplementary Movie 1. Spatial patterning of biological pathways in melanoma.**

**Description:** 100 pathways are shown within the tumor region of sample A. The average expression of the genes annotated for the indicated pathway is shown. Blue: low expression; red: high expression.

**Title: Supplementary Movie 2. Spatial patterning of biological pathways in the melanoma macroenvironment.**

**Description:** 100 pathways are shown within the interface and muscle regions of sample C. The average expression of the genes annotated for the indicated pathway is shown. Blue: low expression; red: high expression.

**Title: Supplementary Data 1**

**Description:** Average log2 fold change of genes upregulated in the interface within the interface, tumor, and muscle SRT clusters.
