# Supplementary material for: Spatially resolved transcriptomics reveals the architecture of the tumor-microenvironment interface
Source: Nat Commun. 2021 Nov 1;12:6278. doi: 10.1038/s41467-021-26614-z (PMC8560802; doi:10.1038/s41467-021-26614-z)
Supplement: Supplementary file 7 — Reporting Summary [file 41467_2021_26614_MOESM7_ESM.pdf]

nature research

Corresponding author(s): Itai Yanai, Richard M. White

Last updated by author(s): September 20, 2021

Reporting Summary

Nature Research wishes to improve the reproducibility of the work that we publish. This form provides structure for consistency and transparency in reporting. For further information on Nature Research policies, see our Editorial Policies and the Editorial Policy Checklist.

Statistics

For all statistical analyses, confirm that the following items are present in the figure legend, table legend, main text, or Methods section.

n/a	Confirmed	
☐	☒	The exact sample size (*n*) for each experimental group/condition, given as a discrete number and unit of measurement
☐	☒	A statement on whether measurements were taken from distinct samples or whether the same sample was measured repeatedly
☐	☒	The statistical test(s) used AND whether they are one- or two-sided<br>*Only common tests should be described solely by name; describe more complex techniques in the Methods section.*
☒	☐	A description of all covariates tested
☐	☒	A description of any assumptions or corrections, such as tests of normality and adjustment for multiple comparisons
☐	☒	A full description of the statistical parameters including central tendency (e.g. means) or other basic estimates (e.g. regression coefficient) AND variation (e.g. standard deviation) or associated estimates of uncertainty (e.g. confidence intervals)
☐	☒	For null hypothesis testing, the test statistic (e.g. F, t, r) with confidence intervals, effect sizes, degrees of freedom and P value noted<br>*Give P values as exact values whenever suitable.*
☒	☐	For Bayesian analysis, information on the choice of priors and Markov chain Monte Carlo settings
☒	☐	For hierarchical and complex designs, identification of the appropriate level for tests and full reporting of outcomes
☐	☒	Estimates of effect sizes (e.g. Cohen's d, Pearson's r), indicating how they were calculated

Our web collection on statistics for biologists contains articles on many of the points above.

Software and code

Policy information about availability of computer code

Data collection	No software was used to collect data.
Data analysis	Custom R, MATLAB, and Python code used for analysis. R version: 3.6.3; MATLAB version: 2019b; Python version: 3.6. Other software/packages used for analysis: Space Ranger v1.0.0, Cell Ranger v5.0.1, Seurat v3.1.4, nichenetR v0.1.0, DoubletFinder v2.0.3, HOMER v4.11. All code used for analysis and plotting available at: doi.org/10.5281/zenodo.5512629

For manuscripts utilizing custom algorithms or software that are central to the research but not yet described in published literature, software must be made available to editors and reviewers. We strongly encourage code deposition in a community repository (e.g. GitHub). See the Nature Research guidelines for submitting code & software for further information.

Data

Policy information about availability of data

All manuscripts must include a data availability statement. This statement should provide the following information, where applicable:

- Accession codes, unique identifiers, or web links for publicly available datasets
- A list of figures that have associated raw data
- A description of any restrictions on data availability

The scRNA-seq, snRNA-seq and SRT data reported in this manuscript have been deposited to the Gene Expression Omnibus under accession number GSE159709. Human scRNA-seq data was obtained from GEO (GSE174401).

Field-specific reporting

Please select the one below that is the best fit for your research. If you are not sure, read the appropriate sections before making your selection.

- [x] Life sciences
- [ ] Behavioural & social sciences
- [ ] Ecological, evolutionary & environmental sciences

For a reference copy of the document with all sections, see nature.com/documents/nr-reporting-summary-flat.pdf

Life sciences study design

All studies must disclose on these points even when the disclosure is negative.

Sample size	Sample size for SRT was determined by the number of arrays available on one SRT slide (n = 3). Sample size for scRNA-seq and snRNA-seq was limited by the number of cells that can be sequenced in one reaction. For microscopy experiments, at least n = 3 technical and biological replicates were performed.
Data exclusions	No data was excluded from the analysis other than quality control filtering.
Replication	SRT, snRNA-seq, and scRNA-seq data has not been replicated due to technical/cost limitations. For microscopy experiments, at least n = 3 technical and biological replicates were performed.
Randomization	No randomization was done as in all cases there was only one experimental group.
Blinding	No blinding was done as in all cases there was only one experimental group.

Reporting for specific materials, systems and methods

We require information from authors about some types of materials, experimental systems and methods used in many studies. Here, indicate whether each material, system or method listed is relevant to your study. If you are not sure if a list item applies to your research, read the appropriate section before selecting a response.

Materials & experimental systems

n/a	Involved in the study	
[ ]	[x]	Antibodies
[ ]	[x]	Eukaryotic cell lines
[x]	[ ]	Palaeontology and archaeology
[ ]	[x]	Animals and other organisms
[x]	[ ]	Human research participants
[x]	[ ]	Clinical data
[x]	[ ]	Dual use research of concern

Methods

n/a	Involved in the study	
[x]	[ ]	ChIP-seq
[x]	[ ]	Flow cytometry
[x]	[ ]	MRI-based neuroimaging

Antibodies

Antibodies used	Primary antibodies used were: goat anti-GFP (abcam #ab5450, 1:200) and mouse anti-acetylated tubulin (Sigma-Aldrich #T6793, 1:100). Secondary antibodies used were: donkey anti-goat IgG conjugated to Alexa 488 (Thermo Fisher Scientific #A11055, 1:250) and goat anti-mouse IgG conjugated to Alexa 555 (Cell Signaling Technology #4409S, 1:250). Hoechst 33342 (Thermo Fisher Scientific #H3570) was added to the secondary antibody solution at 1:1000.
Validation	All antibodies are commonly used in our lab and have been validated by the suppliers. GFP antibody staining was validated by overlap with endogenous GFP fluorescence signal in transgenic reporter lines. Acetylated tubulin staining was validated by overlap with a transgenic cilia reporter.

Eukaryotic cell lines

Policy information about cell lines

Cell line source(s)	ZMEL1 cells: generated in the White lab.
Authentication	The cell line was generated in our lab from a primary zebrafish melanoma so no authentication was required.
Mycoplasma contamination	Mycoplasma does not infect zebrafish cells.
Commonly misidentified lines (See ICLAC register)	No commonly misidentified lines were used in the study.

Animals and other organisms

Policy information about studies involving animals; ARRIVE guidelines recommended for reporting animal research

Laboratory animals	Zebrafish (Danio rerio). Genotype: casper; mitfa-BRAFV600E; p53-/-; mitfa-/-. Age: 6-12 months. Sex was not a variable in the studies and thus was not controlled for. Our transcriptomics data did not allow us to determine the gender of the fish used for our SRT, snRNA-seq and scRNA-seq experiments.
Wild animals	No wild animals were used in the study.
Field-collected samples	No field-collected samples were used in the study.
Ethics oversight	All animal procedures were approved by the Memorial Sloan Kettering Cancer Center Institutional Animal Care and Use Committee (protocol #12-05-008).

Note that full information on the approval of the study protocol must also be provided in the manuscript.
